# *Momoridica charantia* and fermented *Momoridica charantia* with *Leuconostoc mesenteroides* MKSR change intestinal microbial diversity indices and compositions in high-fat and high-cholesterol diet-fed C57BL/6 male mice

**DOI:** 10.3389/fvets.2024.1496067

**Published:** 2024-12-17

**Authors:** Heewon Moon, Kangwook Lee, Jung-Heun Ha, Na Yeun Kim, Hyo Ri Shin, Tae Jin Cho, Nam Su Oh, Jinbong Park, Jingsi Tang, Jae Kyeom Kim, Misook Kim

**Affiliations:** ^1^Department of Food Science and Nutrition, Dankook University, Cheonan, Republic of Korea; ^2^Department of Food Biotechnology, Korea University, Sejong, Republic of Korea; ^3^Department of Pharmacology, College of Korean Medicine, Kyung Hee University, Seoul, Republic of Korea; ^4^State Key Laboratory of Molecular Developmental Biology, Institute of Genetics and Development of Biology, Chinese Academy of Sciences, Beijing, China

**Keywords:** *Momoridica charantia*, fermentation, *Leuconostoc mesenteroides* MKSR, high-fat diet, obesity, gut microbiota

## Abstract

**Introduction:**

This study explores the impact of 4% *Momordica charantia* (MC) and 4% fermented *Momordica charantia* (FMC) on gut microbiota and obesity-related health outcomes in male C57BL/6 mice, a model relevant to veterinary sciences for understanding metabolic and gut health disorders in animals.

**Methods:**

Mice were assigned to four dietary regimens, including control, high-fat and high-cholesterol diet (POS), POS with 4% MC, and POS with 4% FMC (fermented with *Leuconostoc mesenteroides* MKSR) over 12 weeks. Fecal samples were collected for 16S rRNA sequencing to evaluate microbial diversity and composition, key factors influencing animal health.

**Results:**

Both MC and FMC groups exhibited significant alterations in gut microbial communities, with FMC inducing a distinct shift in beta diversity indices. Changes in microbial taxa such as Bacteroidetes, Verrucomicrobia, and Firmicutes were observed, along with enhancement in the ‘L-glutamate and L-glutamine biosynthesis’ pathway. These shifts were associated with reduced body weight gain and liver weights.

**Discussion:**

The findings suggest that MC and FMC have potential benefits for managing diet-induced metabolic disorders and protecting against obesity by modulating gut microbiota and improving gut metabolism.

## 1 Introduction

The World Health Organization defines obesity as a medical condition in which an abnormal or excessive amount of fat is accumulated in adipose tissue to the extent of harming health. In clinical practice, it is specifically defined as having a body mass index (BMI) of 30 kg/m^2^ or higher (1). Over the past 50 years, the prevalence of obesity has approximately tripled worldwide, affecting individuals of all age groups regardless of race or social status (2). Many studies have demonstrated that this condition is associated with metabolic diseases (e.g., type 2 diabetes) (3), cardiovascular diseases (4, 5), arthritis (6, 7), and neurodegenerative diseases [e.g., Alzheimer’s disease (8)], causing complications in nearly every organ in the human body. Therefore, obesity is a major health disease that affects life expectancy and medical costs (9). With more than 300 million people worldwide having a BMI of 30 kg/m^2^, and projections suggesting that approximately 20% of the world’s population will be obese by 2030, obesity has become a global public health issue. To prevent the risks of metabolic diseases, effective preventive strategies are warranted.

Many previous studies have shown that the gut microbiome is related to obesity (10, 11). The gut microbiome can potentially regulate obesity development by altering host metabolism and energy utilization (12). Since gut microbes have been associated with the progression of obesity (13), substituting a microbial population (“bad” microbes) that promotes obesity with a microbial population that cultivates a healthy state (“good” microbes) may offer viable treatment and preventive strategies (14). Studies have shown that individuals with obesity and type 2 diabetes exhibit an imbalance in their gut microbiome (15, 16). With respect to specific mechanisms, imbalanced gut microbiota disrupts the integrity of intestinal barriers and functions of gut-associated lymphoid tissues. This disruption allows lipopolysaccharides, which are structural components of bacteria, to penetrate and activate inflammatory pathways, ultimately leading to inflammation (17). Furthermore, the presence of cytokines, triggered by inflammatory pathways, contributes to the development of insulin resistance through changes in insulin receptor signaling. In addition, it can increase the production of the ghrelin hormone, which is associated with satiety, leading to increased food intake and contributing to self-maintenance cycles (17). Therefore, lipid catabolism may be suppressed, promoting increased body fat (17). In recent years, research has explored the modulation of the gut microbiome using natural substances as a means to mitigate and/or prevent obesity. For instance, Yanan et al. (18) reported that controlling the gut microbiome using the lotus leaf (*Nelumbo nucifera*) extract was effective in reducing body weight in mice. Other studies have reported similar results using different natural substances, such as *Artemisia argyi* leaf (19), safflower (20), and *Rosmarinus officinalis* (21).

*Momordica charantia* (MC), also known as bitter melon, is a tropical and subtropical edible plant, which is widely used as a traditional functional food. MC grows naturally in East Asia and is found in natural environments such as forests, streams, and rivers. MC contains various antioxidants and offers preventive benefits against obesity and dyslipidemia (22–24). Specifically, charantin, a major functional compound of MC, reduces insulin resistance by increasing cellular glucose uptake and promoting hepatic and muscular glycogen synthesis (25–27). In our previous study, we demonstrated that both non-fermented MC and fermented MC (FMC) significantly mitigated metabolic complications induced by a high-fat and high-cholesterol diet in mice (POS), including lower body weight, reduced liver fat, and decreased levels of serum lipids (24). Although these findings highlighted the overall metabolic benefits of MC and FMC, the specific role of gut microbiota modulation in these effects remained unexplored. Therefore, this study aims to investigate the effects of MC and FMC on gut microbiota and obesity-related health outcomes in high-fat-cholesterol diet-fed C57BL/6 male mice, focusing on microbial diversity and key metabolic pathways.

## 2 Materials and methods

### 2.1 Animal study

The experimental design and dietary interventions followed the protocol described in our previous study (24). Briefly, 40 male C57BL/6 mice were randomly assigned to four groups: NEG (mice fed with the control diet of 7% soybean oil; wt:wt), POS (mice fed with 14.5% of the high-fat and high-cholesterol diet; wt:wt), 4% MC, and 4% FMC. The compositions of the experimental diets are detailed in Table 1.

**Table 1:** Composition of the experimental diets.

Diet composition (g)	NEG^a^	POS^b^	4% MC^c^	4% FMC^d^
Casein	200	200	200	200
L-cystine	3	3	3	3
Sucrose	100	90	90	89.04
Corn starch	397.486	259.49	259.49	259.49
Dextrose	132	125	125	121.49
Lard		145	145	145
Soybean oil	70	70	70	70
Cholesterol	0	10	10	10
Cellulose	50	50	10	14.46
Mineral mix	35	35	35	35
Choline bitartrate	2.5	2.5	2.5	2.5
Vitamin mix	10	10	10	10
t-Butylhydroquinone	0.014	0.014	0.014	0.014
MC			40	
FMC				40
Total	1000.00	1000.00	1000.00	1000.00

a NEG, mice fed with the control diet (7% soybean oil; wt:wt).

b POS, mice fed with the high-fat and high-cholesterol (HFCD) diet (14.5%; wt:wt) and cholesterol (1%; wt:wt).

c 4% MC, mice fed with the HFCD diet with 4% MC (wt:wt).

d 4% FMC, mice fed with the HFCD diet with 4% FMC (wt:wt).

MC, *Momoridica charantia*; FMC, Fermented *Momoridica charantia*, which is inoculated with *Leuconostoc mesenteroides* MKSR.

Ethical approval for all animal care and experimental procedures was granted by the Institutional Animal Care and Use Committee (IACUC) of Dankook University (IACUC Approval number: DKU-22-025).

### 2.2 Feces collection and sample preparation for 16S rRNA sequencing

Fresh mouse feces were collected from the cecum, stored in sterile tubes, rapidly frozen on dry ice, and then transferred to an −80°C cryogenic freezer for cryopreservation until DNA extraction (28). Microbial DNA was extracted from 32 samples (n = 8 per each group) by using the ^®^ PowerFecal^®^ Pro DNA Kit (Qiagen, Hilden, Germany), following the manufacturer’s protocol.

While our previous study used 10 animals per group (24), in this study, only 8 samples per group were selected for 16S rRNA sequencing. This change was made to optimize resources and focus on high-quality data. The selection of these 8 samples was based on sample quality control results, with samples chosen to ensure sufficient DNA yield and integrity. The 8 samples that passed QC were used for sequencing.

### 2.3 16S rRNA sequencing, quality control, and library construction

For the purpose of quality control, the samples from the extracted DNA were amplified. During the amplification process, the V3–V4 hypervariable regions were amplified and subsequently run on a 2% agarose gel at 40 V for 40 min (Supplementary Figure S1). Following this step, equal quantities of PCR products from each sample were combined and subjected to end-repair, A-tailing, and ligation with Illumina adapters. The libraries were then sequenced using a paired-end Illumina platform, as described by Schmidt et al. (29). Subsequently, the sequence error rate was calculated using the Phred score, where Phred scores of 10, 20, 30, and 40 correspond to error rates of 10, 1, 0.1, and 0.01%, respectively. The Phred scores of all samples are presented in Supplementary Figures S2A–D.

### 2.4 Statistical and bioinformatics analyses

Phenotypic and biochemical endpoints are presented as means ± standard deviations. Before comparisons, the Shapiro–Wilk test was conducted to assess normality. Subsequently, Bartlett’s test was used to verify the homogeneity of the variances. All endpoint markers passed the normality and the homogeneity of variance tests before conducting one-way analysis of variance (ANOVA). ANOVA was employed to determine statistical significance between the groups; when significant differences were detected, Duncan’s *post-hoc* test was used to further assess between-group differences. A *p*-value of less than 0.05 was considered statistical significant. Any group means that did not share the same letter were deemed significantly different. Statistical evaluations were performed using GraphPad Prism 9 (GraphPad Software, San Diego, CA, United States).

For the microbiota results, alpha diversity was calculated using amplicon sequence variants (ASVs), Chao1, abundance-based coverage estimators (ACE), as well as Shannon and Simpson diversity indices. On the other hand, beta diversity was determined using the Bray–Curtis index, the Jaccard index, the weighted UniFrac distance, and the unweighted UniFrac distance, and it was visualized using principal coordinate analysis (PCoA). Taxonomic composition was visualized using relative abundance, calculated as the total number of individual species divided by the total number of species population, and then multiplied by one hundred. Venn analysis was used to visualize the unique and shared ASVs (or compositional membership) among the groups, making it suitable to show the core microbiome and the impact/shift induced by the sample interventions (i.e., 4% MC and 4% FMC). Linear discriminant analysis effect size (LefSe) was calculated using the following parameters: a differential significance threshold of 0.05, a p-adjust_method of false discovery rate, a sample number ratio of 0.6667 for each bootstrap, and a total of 30 bootstrap tests. This was used to identify key features of each group. A Linear Discriminant Analysis (LDA) score (Log 10) greater than 1 was considered a meaningful discriminative biomarker. Following the LefSe, the taxa of bacteria with statistically significant changes (*p* < 0.05) in the relative abundance among the groups were compared using a non-parametric factorial Kruskal–Wallis (KW) sum rank test. Random Forest analysis was conducted using the following parameters: a differential significance threshold of 0.05, a p_adjust_method of FDR, a sample number ratio of 0.6667 for each bootstrap, a total of 30 bootstrap tests, and ntree set to 1,000. This analysis was used to identify key taxonomic features of each group. Pathway abundances were calculated based on the abundances of gene families that can be linked to reactions within pathways using PICRUSt2.^1^

## 3 Results

In our previous study, *Momordica charantia* (MC) and fermented MC (FMC) were shown to significantly mitigate diet-induced obesity in C57BL/6 mice subjected to a high-fat, high-cholesterol diet (HFCD). The focus of our previous study was to establish the efficacy of these interventions in reducing key obesity-related phenotypes, including weight gain, final body and liver weight, and white adipose tissue accumulation. The results revealed that both 4% MC and 4% FMC interventions led to distinct improvements in these parameters when compared to the POS group, with MC showing particularly significant reductions in final body weight and total weight gain (Table 2). Based on these findings, the current study specifically selected the 4% MC and 4% FMC groups for a detailed analysis of gut microbiota composition and diversity as these groups exhibited the most promising anti-obesity effects. The current study aimed to explore the underlying microbial mechanisms that may contribute to the observed phenotypic changes. By focusing on the gut microbiota, we sought to elucidate how these dietary interventions modulate microbial communities and potentially influence metabolic health outcomes.

**Table 2:** Weight gain, food intake, tissue weights, and liver toxicity markers of the mice after the 12-week intervention.

Measures	NEG^a^ (*n* = 10)	POS^b^ (*n* = 10)	4%MC^c^ (*n* = 10)	4%FMC^d^ (*n* = 10)
Final weight (g)	28.4 ± 1.9	37.5 ± 2.9^*^	34.2 ± 2.2^#^	34.9 ± 2.8^#^
Total weight gain (g)	13.4 ± 2.0	22.5 ± 2.3^*^	19.3 ± 1.8^#^	19.9 ± 2.4
Food intake (g)	9.2 ± 1.3	9.7 ± 1.4	9.5 ± 1.3	9.1 ± 1.1
Liver weight (g)	1.0 ± 0.1	1.5 ± 0.2^*^	1.4 ± 0.2	1.4 ± 0.2
Epididymal fat (g)	0.32 ± 0.1	1.23 ± 0.3^*^	1.10 ± 0.4	0.87 ± 0.3
Retroperitoneal fat (g)	0.10 ± 0.04	0.35 ± 0.08^*^	0.32 ± 0.14	0.25 ± 0.12^#^
AST (IU/dL)	2.56 ± 0.48	3.18 ± 0.33^*^	3.06 ± 0.33	2.79 ± 0.24
ALT (IU/dL)	1.02 ± 0.27	2.37 ± 1.07^*^	1.74 ± 0.88	1.33 ± 0.69

Data are expressed as mean ± standard deviation.

*denotes *p* < 0.05, compared to the NEG group. ^#^denotes *p* < 0.05, compared to the POS group.

a NEG, mice fed with the control diet (7% soybean oil; wt:wt).

b POS, mice fed with the high-fat and high-cholesterol (HFCD) diet (14.5%; wt:wt) and cholesterol (1%; wt:wt).

c 4% MC, mice fed with the HFCD diet with 4% MC (wt:wt).

d 4% FMC, mice fed with the HFCD diet with 4% FMC (wt:wt).

MC, *Momoridica charantia*; FMC, Fermented *Momoridica charantia*, which is inoculated with *Leuconostoc mesenteroides* MKSR; ALT, Alanine aminotransferase; AST, Aspartate aminotransferase.

### 3.1 FMC intervention changed the alpha diversity indices in the gut microbiota

The assessment of QC data for extracted DNA and library construction entails a comprehensive evaluation, taking into account the necessary sample quality requirements for library preparation and sequencing. For amplification, the hypervariable regions V3-4 were amplified and then subjected to run on a 2% agarose gel at 40 V run over 40 min, as shown in Supplementary Figure S1. Subsequently, equal amounts of PCR products from each sample were pooled, end-repaired, A-tailed, and then ligated with Illumina adapters. The libraries were sequenced on a paired-end Illumina platform to generate 250 bp paired-end raw reads. The sequence error rate was then calculated using the Phred score. The relationship between the sequencing error rate (e) and the sequencing base quality value (i.e., Phred) is presented in Supplementary Table S1. The sequencing error rate of the examined samples at 150 base pair reading is shown in the Supplementary Figure S2. As shown in the figure, all samples showed Phred scores around 35, thus passing the QC test for the sequencing error rate.

Alpha diversity is applied to the analysis of microbial community diversity within a sample; alpha diversity indices reflect the diversity of a single sample, where the richness and evenness of microbial communities are presented (30). More specifically, species richness is the count of the number of species present in a sample. It does not consider the abundance of species or their relative distributions. On the other hand, species evenness is a measure of the relative abundance of different species that make up the richness.

**Figure 1:**
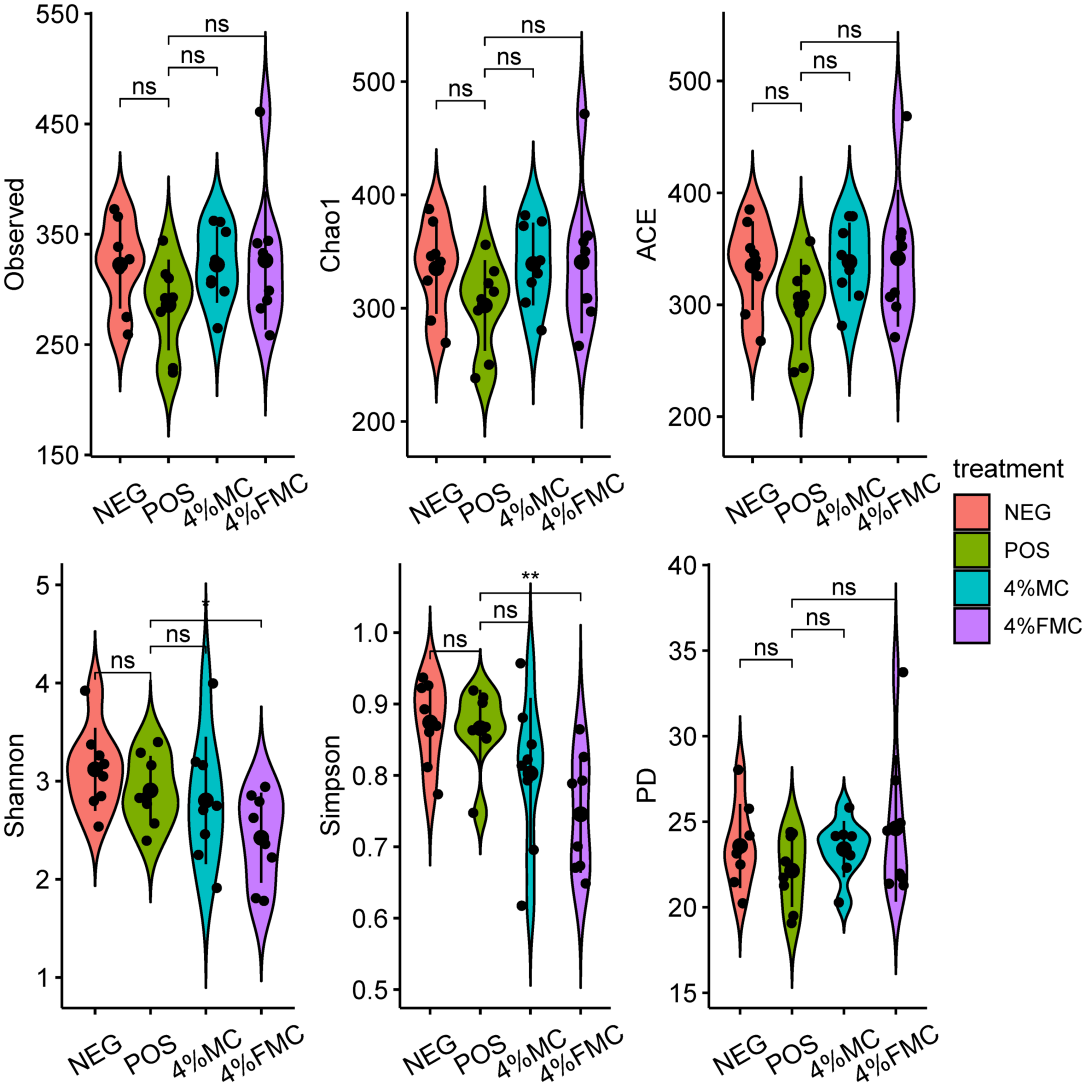
Alpha diversity indices demonstrated the effects of 4% FMC on the gut microbiome. Alpha diversity was assessed using the Observed ASVs, Chao1, ACE, Shannon’s, Simpson’s, and PD indices. For statistical comparisons, a *t*-test (reference group was the POS) was used to examine the differences between the groups. The black dots within the violin represent the individual values of the respective groups. The bar line within the violin represent the standard deviation. “ns,” *p*-value > 0.05; “*,” *p*-value < 0.05, and “**,” *p*-value < 0.01. NEG, mice fed with the control diet (7% soybean oil; wt:wt); POS, mice fed with the high-fat and high-cholesterol (HFCD) diet (14.5%; wt:wt) and cholesterol (1%, wt:wt); 4% MC, mice fed with the HFCD diet with 4% *Momoridica charantia* (wt:wt); and FMC, mice fed with the HFCD diet with 4% fermented *Momoridica charantia* (wt:wt), which was inoculated with *Leuconostoc mesenteroides* MKSR.

In the present study, we used several indices to investigate the samples’ alpha diversity. More specifically, the Observed refers to the total number of ASVs in a sample and serves as the simplest measure of alpha diversity. Chao1 and ACE are abundance-based estimators used for estimating species richness. The higher the values of these two indices, the greater the number of observed taxonomic units (OTUs). In the study, we found no difference in the Observed ASVs, Chao1 index, and ACE (Abundance-based coverage estimators) index, as shown in Figure 1 (upper panels, from left to right).

Shannon’s index considers both species richness and species evenness in the distribution of a sample. The value of Shannon’s index increases as diversity increases. Simpson’s index accounts for the proportion of species in a sample. It increases as both the richness and evenness of the community increase. Phylogenetic diversity (PD) is unlike the diversity measures mentioned above; the PD measure incorporates information from phylogenetic relationships represented in phylogenetic trees among species within a sample. Faith’s PD is calculated as the sum of the branch lengths of all species in a sample. In the present study, the values of the Shannon’s index (*p* < 0.05) and Simpson’s index (*p* < 0.01) were lower in the 4% FMC compared to the POS group. No differences were observed in the PD index (Figure 1). Given the definitions of the Shannon’s and Simpson’s indices, it is reasonable to conclude that the administration of 4% FMC impacted both species richness and evenness in the gut microbial samples.

The observed decrease in both Shannon’s and Simpson’s indices in the 4% FMC group as compared to the POS group indicates a notable shift in the gut microbial community structure, potentially influencing species richness and evenness. It is imperative to explore the underlying mechanisms by which 4% FMC administration has led to these alterations and understand the specific microbial taxa affected. Given that the PD index showed no significant difference, it suggests that although the community structure was impacted in terms of richness and evenness, the overall phylogenetic diversity remained stable. This discrepancy between the diversity indices raises questions about the selective impact of 4% FMC on certain microbial taxa, possibly favoring some while inhibiting others, without altering the broader phylogenetic relationships within the community.

### 3.2 Both MC and FMC interventions changed the beta diversity indices in the gut microbiota

**Figure 2:**
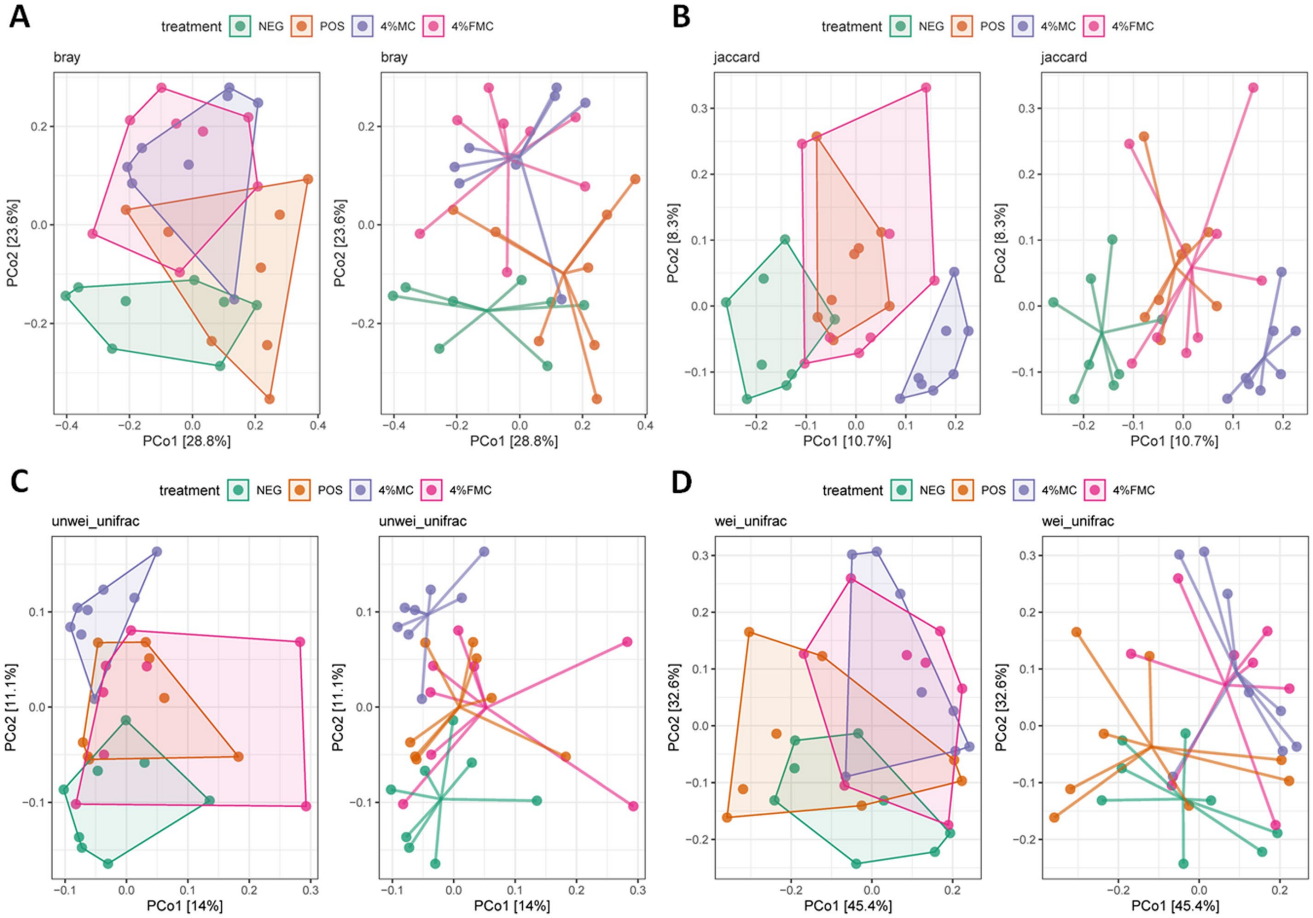
PCoA plots of beta diversity in the gut microbiome. (A) Bray–Curtis index, (B) Jaccard index, (C) unweighted UniFrac distance, and (D) weighted UniFrac distance were generated using PCoA analyses. NEG, mice fed with the control diet (7% soybean oil; wt:wt); POS, mice fed with the high-fat and high-cholesterol (HFCD) diet (14.5%; wt:wt) and cholesterol (1%; wt:wt); 4% MC, mice fed with the HFCD diet with 4% *Momoridica charantia* (wt:wt); and FMC, mice fed with the HFCD diet with 4% fermented *Momoridica charantia* (wt:wt), which was inoculated with *Leuconostoc mesenteroides* MKSR.

Beta diversity assesses dissimilarities between samples, measuring differences between samples from different groups to identify if there are any differences in the overall community composition and structure (30). The most popular beta diversity measures in microbiome research include the Bray–Curtis index (for compositional data), the Jaccard index (for presence/absence data, ignoring abundance information), the unweighted UniFrac distance (takes into account the phylogenetic tree information), and the weighted UniFrac distance (provides an abundance-weighted version of the unweighted UniFrac distance). These distances can then be subjected to ordination. Ordination is a common concept in ecology that reduces the dimensionality of data for further evaluation or visualization. PCoA is a non-linear dimension reduction technique used to visualize sample similarity. The two most informative top components (PCo1 and PCo2) are visualized in a 2-dimensional plot. In Figure 2, PCoA plots for the Bray–Curtis index, the Jaccard index, the unweighted UniFrac distance, and the weighted UniFrac distance are provided, offering an intuitive visualization (Figure 2).

**Figure 3:**
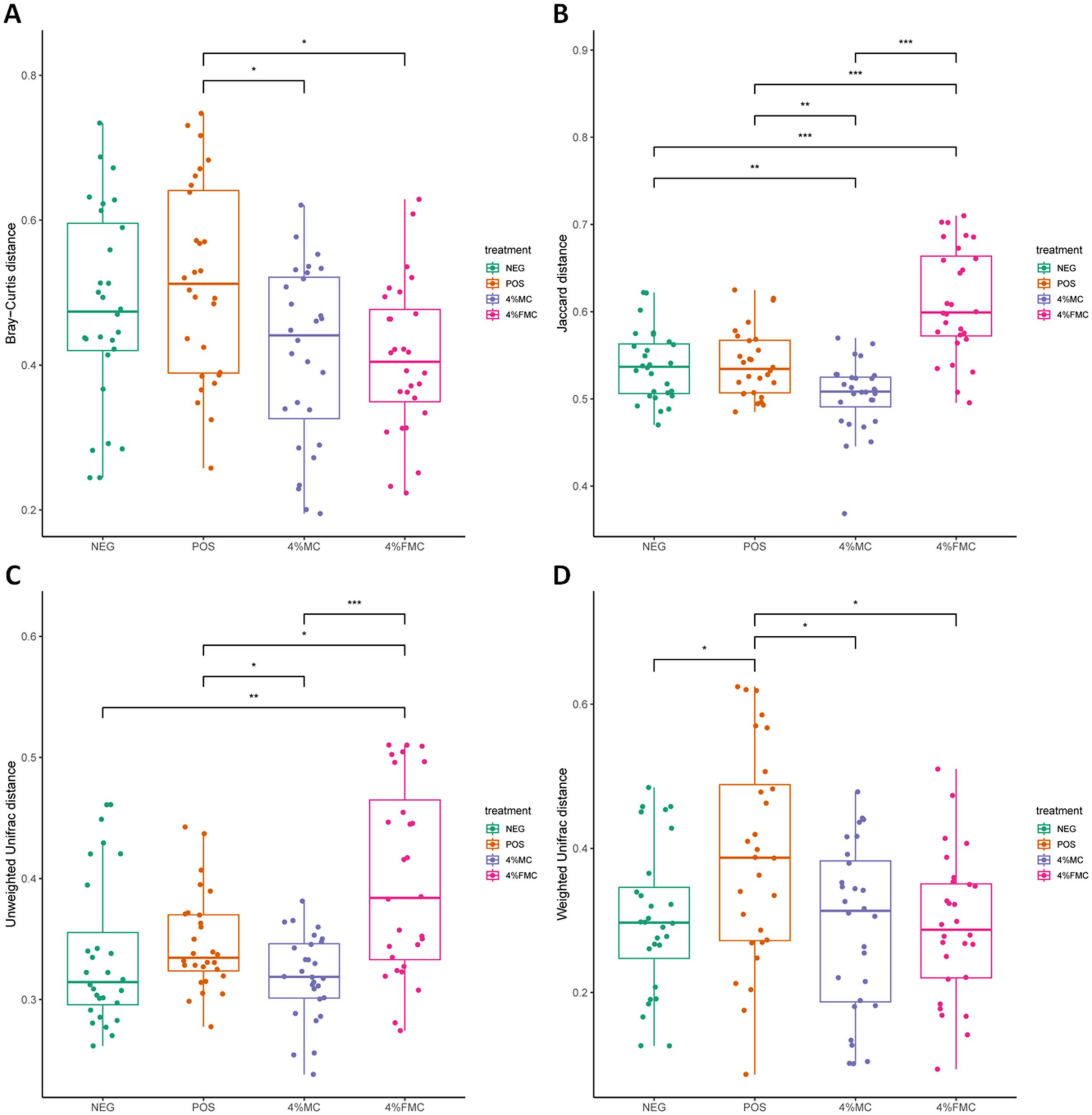
Statistical analysis of the beta diversity indices in gut microbiome. (A) Bray–Curtis index, (B) Jaccard index, (C) unweighted UniFrac distance, and (D) weighted UniFrac distance. Wilcoxon rank-sum test was used in the differential test of distances within/between the groups. The *p*-value is adjusted by FDR. “*,” *p*-value < 0.05; “**,” *p*-value < 0.01; and “***,” *p*-value < 0.001. NEG, mice fed with the control diet (7% soybean oil; wt:wt); POS, mice fed with the high-fat and high-cholesterol (HFCD) diet (14.5%; wt:wt) and cholesterol (1%; wt:wt); 4% MC, mice fed with the HFCD diet with 4% *Momoridica charantia* (wt:wt); FMC, mied fed with the HFCD diet with 4% fermented *Momoridica charantia* (wt:wt), which is inoculated with *Leuconostoc mesenteroides* MKSR.

Although the ordination figures above provide visual insights, further statistical analyses are warranted. First, the Bray–Curtis distance was examined and compared through pairwise comparisons, in which both 4% MC and 4% FMC groups had a significant difference compared to the POS group (*p* < 0.05 for both; Figure 3A). Specifically, the Bray–Curtis distance was decreased in the intervention groups. In the Jaccard distance analysis, the 4% MC group showed a lower index value, while the 4% FMC group showed a greater index value than the other groups (Figure 3B). In the unweighted UniFrac distance analysis, similar to the Jaccard distance, the 4% MC group exhibited a lower value, while the 4% FMC group exhibited a greater distance (*p* < 0.05 for both; Figure 3C). Finally, in the weighted UniFrac distance index, both 4% MC and 4% FMC groups exhibited lower values than the POS, which is consistent with the Bray–Curtis distance result (*p* < 0.05 for both; Figure 3D).

The findings derived from the Bray–Curtis distance indicate a significant shift in the microbial community composition within both 4% MC and 4% FMC groups as compared to the POS group. The decrease in the Bray–Curtis distance in the intervention groups suggests a convergence in the community structure, raising questions about the specific microbial taxa influenced and the resultant alterations in ecological interactions within the gut microbiota. The observed disparity in the Jaccard and unweighted UniFrac distances between the 4% MC and 4% FMC groups highlights the differential impact of these interventions on community membership and phylogenetic diversity. The lower values in the 4% MC group indicate a potential increase in shared species, whereas the higher values in the 4% FMC group suggest a divergence in microbial composition. Consistent with the Bray–Curtis distance results, the lower values in the weighted UniFrac distance for both intervention groups compared to the POS group suggest alterations in the abundance of phylogenetically related microbial taxa. Furthermore, the contrasting patterns observed in different distance measures call for an integrated analysis of the compositional and phylogenetic shifts observed in the 4% MC and 4% FMC groups.

### 3.3 Taxonomic composition signatures were changed in the MC and FMC intervention groups

**Figure 4:**
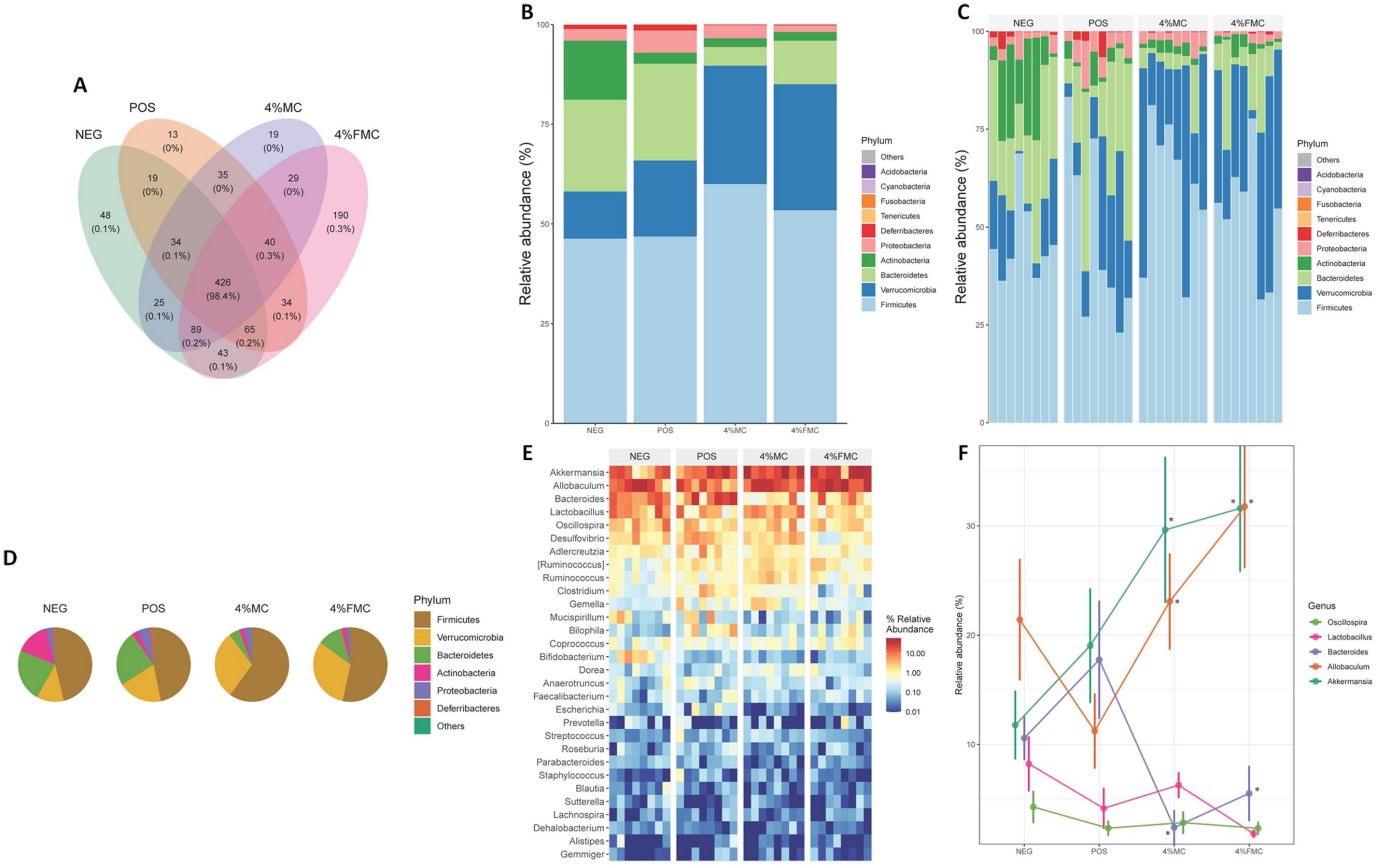
Altered taxonomic composition signatures in the 4% MC and 4% FMC intervention groups. (A) Venn analysis visualizes shared and unique ASVs in each group, (B) Relative abundance of the top 10 phyla and effects of sample administration on the gut microbiome in each group, (C) Relative abundance of the top 10 phyla and effects of sample administration on the gut microbiome in individual mouse, (D) Relative abundance of the top six phyla and effects of sample administration on the gut microbiome in each group, (E) Heatmap plot for the top 30 taxa at the genus level, and (F) Relative abundance of the most enriched top five taxa at the genus level. The *p*-value is adjusted by FDR. “*,” *p*-value < 0.05. NEG, mice fed with the control diet (7% soybean oil; wt:wt); POS, mice fed with the high-fat and high-cholesterol (HFCD) diet (14.5%; wt:wt) and cholesterol (1%; wt:wt); 4% MC, mice fed with the HFCD diet with 4% *Momoridica charantia* (wt:wt); and FMC, mied fed with the HFCD diet with 4% fermented *Momoridica charantia* (wt:wt), which was inoculated with *Leuconostoc mesenteroides* MKSR.

First, the Venn analysis serves as a valuable tool for elucidating both the unique and shared ASVs across different groups, thereby offering insights into the core microbiome and the alterations induced by sample interventions. In this context, the represented integers indicate the number of ASVs, while the accompanying percentage data reflect the proportion of the sequence number to the total sequence number. According to the conducted Venn analysis, a substantial overlap was observed with a total of 426 ASVs shared across all groups (Figure 4A). In addition, distinct variations were noted, with 190 ASVs and 19 ASVs exclusively detected in the 4% FMC and 4% MC groups, respectively. These findings highlight the distinct microbial compositional membership within each group and suggest the potential impact of the respective interventions on microbial diversity.

Relative abundance (the total number of individual species divided by the total number of species population, multiplied by one hundred) is calculated to show what percentage of the microbiome is comprised of a specific taxon. The first bar plot visualizes the mean relative abundance for each group using the top 10 phyla (Figure 4B). The next bar plot visualizes the relative abundance for each sample (within respective groups) using the top 10 phyla (Figure 4C). Next, a pie chart was prepared using the mean relative abundance of the top six phyla for each group (Figure 4D). Overall, dramatic decreases in Bacteroidetes were observed in both intervention groups, while Verrucomicrobia increased in the 4% MC and 4% FMC groups (Figures 4E,F). Similarly, Firmicutes increased in both intervention groups, with varying magnitudes. As shown in Figure 4C, there were individual variations between the mice, but the overall trends within each group remained consistent. On the other hand, the heatmap displays the relative abundance of the top 30 taxa at the genus level among the groups (Figure 4E). The top 30 genera presented in the heat map were further narrowed down to the top five genera, which are displayed in Figure 4F. Briefly, *Bacteroides* decreased in the intervention groups compared to the POS, while *Allobaculum* and *Akkermansia* were increased by the samples (*p* < 0.05 for all; Figure 4F).

### 3.4 Microbial community differences and predicted biological functions were changed in the MC and FMC intervention groups

**Figure 5:**
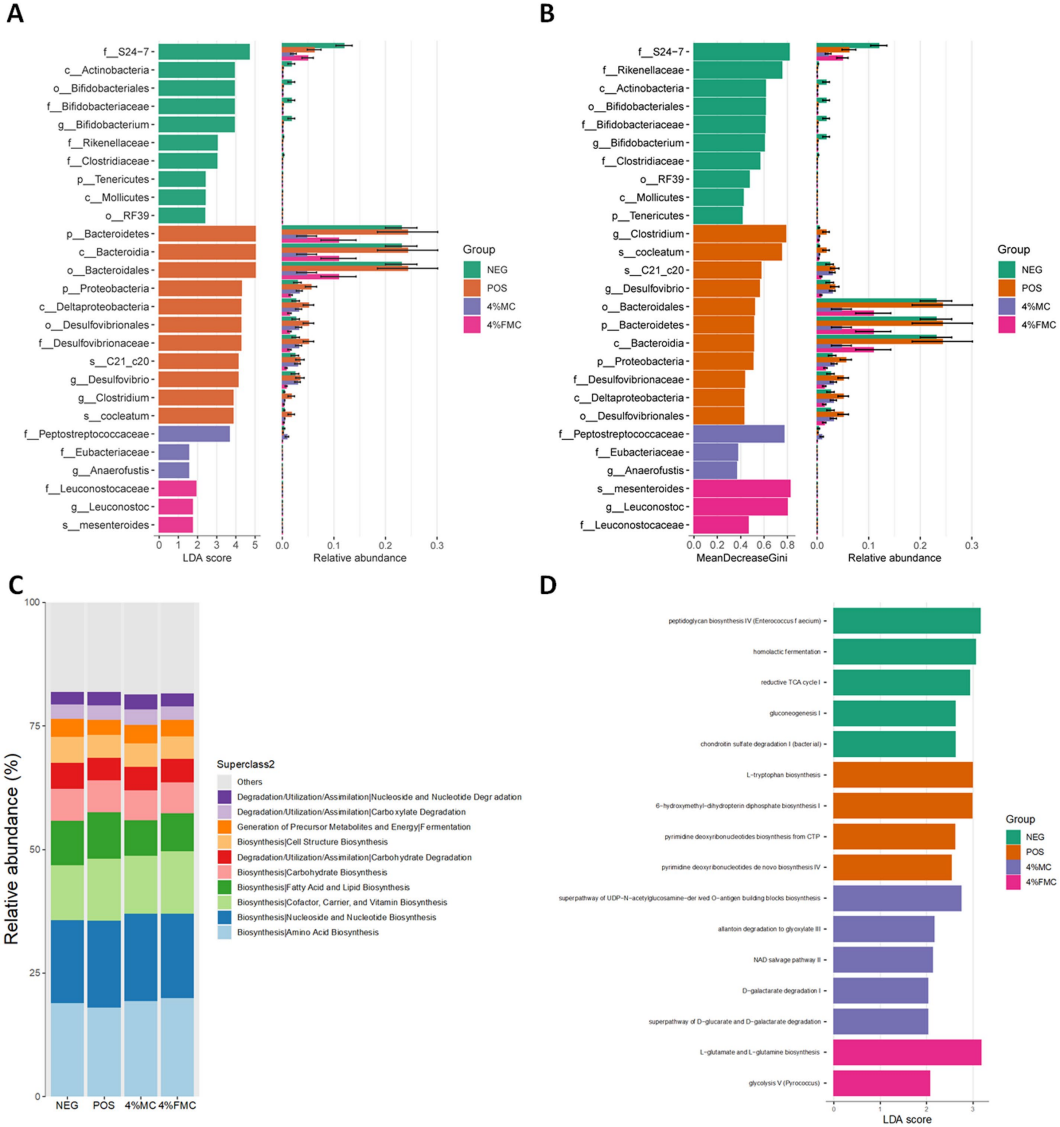
Differential analysis of the taxonomic abundance and biological function prediction. (A) LefSe analysis showed key features of each group, (B) Random Forest analysis showed key taxonomic features of each group, (C) MetaCyc pathways predicted biological function enriched in each group based on relative abundance, and (D) MetaCyc pathways predicted biological function enriched in each group based on LefSe. NEG, mice fed with the control diet (7% soybean oil; wt:wt); POS, mice fed with the high-fat and high-cholesterol (HFCD) diet (14.5%; wt:wt) and cholesterol (1%; wt:wt); 4% MC, mice fed with the HFCD diet with 4% *Momoridica charantia* (wt:wt); and FMC, mied fed with the HFCD diet with 4% fermented *Momoridica charantia* (wt:wt), which was inoculated with *Leuconostoc mesenteroides* MKSR.

The differential abundance test is used to identify significant taxa in determining the community differences across groups. Two methods, LefSe and Random Forest, are used to capture the important characteristics. The LefSe is an algorithm for high-dimensional biomarker discovery that identifies taxa characterizing the differences between two or more biological conditions (31). Figure 5A shows these taxa with the LDA score (log 10) > 1, where the horizontal bars represent the effect size for each taxon. The length of the bar represents the log10 transformed LDA score. The threshold for discriminative features on the logarithmic LDA score is usually set to 2.0. The taxon of bacteria with a statistically significant change (*p* < 0.05) in the relative abundance among the groups was compared; a few key features were noted. First, both 4% MC and 4% FMC groups had a lower relative abundance of the phylum Bacteroidetes, which might have been influenced by the order Bacteroidales (Figure 5A); other features are also shown in Figure 5A, but their relative abundance was somewhat negligible. Notably, in the 4% FMC group, *Leuconostoc mesenteroides* was found to be a key feature, which is consistent, given the preparation process of FMC (i.e., the inoculation of *Leuconostoc mesenteroides* MKSR KCTC 15665P). On the other hand, the Random Forest analysis was performed to compute importance scores (mean decreasing Gini index, MeanDecreaseGini) for estimating the significance of variables. The Random Forest analysis bar plot presents similar patterns to those seen in the LefSe analysis (Figure 5B) with varying orders, as determined by MeanDecreaseGini (Figure 5B).

Pathway abundances are calculated based on the abundances of gene families that can be linked to reactions within pathways. MetaCyc pathways were predicted using the PICRUSt2, which are shown in Figure 5C. Overall, no dramatic difference was noted between the groups. Since the MetaCyc pathway is based on relative abundance, the next prediction was conducted using the LefSe method; in other words, group characteristics were determined based on the LDA score. Different pathways enriched in each group are shown in Figure 5D. Among the pathways, the most significantly enriched pathway was “L-glutamate and L-glutamine biosynthesis” in the 4% FMC group (Figure 5C).

The significant influence of dietary factors on gut microbiota composition and, subsequently, on host health and disease susceptibility has garnered significant interest in recent years. In the present study, we investigated the impacts of 4% MC and 4% FMC interventions on diet-induced obesity phenotypes and their subsequent effect on gut microbiota. Initial analyses centered on diversity indices indicated that the 4% FMC intervention induced noticeable shifts in gut microbial community structure, potentially influencing species richness and evenness. Despite changes in the community structure, overall phylogenetic diversity remained somewhat constant, as indicated by the unaltered PD index. This inconsistency between diversity metrics raised questions regarding the selective influence of 4% FMC on particular microbial taxa, highlighting a possible preferential enhancement of certain microbes over others, without majorly altering the overarching phylogenetic relationships within the community. When examining the beta diversity indices, both interventions showcased significant variations in the gut microbiota. The Bray–Curtis distance revealed a notable shift in microbial community composition, implying convergence in community structure for intervention groups. This raises further questions about the specific taxa influenced and the subsequent alterations in ecological interactions within the gut microbiota. Meanwhile, the distinct patterns in the Jaccard and unweighted UniFrac distances between the 4% MC and 4% FMC groups underscored the differential effects of the interventions on community membership and phylogenetic diversity.

## 4 Discussion

The relationship between specific bacterial phyla and dietary factors was a crucial aspect of our investigation. Regarding Bacteroidetes, studies have shown that animal-based diets increase the abundance of bile-tolerant microorganisms (including Bacteroidetes) while decreasing the levels of Firmicutes that metabolize dietary plant polysaccharides (32). These observations were recapitulated in our study, where, at the phylum levels, the relative abundance of Firmicutes was greatly increased in the 4% MC and 4% FMC groups (Figure 4D). In line with this finding, at the genus level, the relative abundance of *Bacteroides* (a subset of phylum Bacteroidetes) increased in the POS group, while it decreased in the 4% MC and 4% FMC groups (Figure 4F). However, a hasty conclusion should be avoided as there are inconsistencies in the association between Bacteroidetes, Firmicutes, and obesity across different studies, indicating the complexity of the relationship (33).

As shown in Figure 5C, the “L-glutamate and L-glutamine biosynthesis” pathway was significantly more enriched in the 4% FMC group, suggesting several potential physiological implications. First, L-glutamine is considered a crucial nutrient for the health and maintenance of the intestinal mucosa. Enhanced biosynthesis of L-glutamine by the gut microbiota can support gut barrier function and reduce the permeability of the gut, which is sometimes referred to as “leaky gut” (34). In the context of obesity, a few amino acids serve as substrates for short-chain fatty acids; specifically, glutamate can be metabolized to acetate and butyrate, both of which play a role in the development of obesity (35). Together, these predictions should be further validated and interpreted carefully in the context of obesity to understand the health-promoting effects of MC and FMC.

The possible reason for the significant enrichment of the ‘L-glutamate and L-glutamine biosynthesis’ pathway in the 4% FMC group compared to the MC group likely stems from the fermentation process involving *Leuconostoc mesenteroides* (37). Fermentation can enhance microbial metabolic activity, including amino acid biosynthesis, by altering the gut microbiota composition. *Leuconostoc mesenteroides* is known to produce lactic acid and other metabolites that can support the biosynthesis of glutamate and glutamine (38). These amino acids play crucial roles in gut health, immune regulation, and energy metabolism, which may explain why their biosynthesis pathways were more enriched in the FMC group (36). The fermentation process could have facilitated the production of these bioactive compounds, leading to enhanced metabolic outcomes compared to the non-fermented MC group.

Furthermore, FMC, in particular, shows promise in modulating gut microbiota and improving metabolic health, which could be utilized in developing functional foods or supplements aimed at reducing obesity and its associated metabolic disorders. These findings suggest that FMC could be an effective component in dietary strategies for preventing or managing obesity.

Delving into taxonomic compositions, the Venn analysis revealed a significant overlap of the ASVs across groups, providing insights into the core microbiome and the effects of the sample interventions. The alterations in the relative abundance of specific taxa, such as Bacteroidetes, Verrucomicrobia, and Firmicutes, brought to the forefront the unique microbiota landscapes carved by the interventions. Notably, the genus-level analyses highlighted the significant variations in taxa such as *Bacteroides*, *Allobaculum*, and *Akkermansia* due to the interventions. To understand the functional implications of these microbial shifts, pathway predictions based on the MetaCyc database were made. One particularly intriguing observation was the enrichment of the “L-glutamate and L-glutamine biosynthesis” pathway in the 4% FMC group. Considering the importance of L-glutamine in gut barrier maintenance and its metabolic role in obesity, this finding paves the way for further investigation into the therapeutic potential of FMC in obesity management.

## 5 Conclusion

In summary, the study emphasizes the pivotal role of dietary interventions, specifically MC and FMC, in modulating gut microbiota compositions and their subsequent implications in diet-induced obesity phenotypes. Our findings represent one of the first demonstrations of the distinct metabolic effects of FMC on gut microbiota, particularly through the enhancement of the ‘L-glutamate and L-glutamine biosynthesis’ pathway. This unique modulation of gut microbiota by FMC offers novel insights into its potential as a functional dietary intervention for obesity and related metabolic disorders. The observed microbial shifts and associated functional pathways provide valuable insights into potential strategies for addressing obesity and related metabolic disorders. However, it is imperative to validate these findings in larger cohorts and integrate them with comprehensive metabolic and physiological datasets for a holistic understanding of the health implications of MC and FMC interventions. Future research should focus on long-term clinical trials to assess the efficacy of FMC in human populations, as well as exploring the mechanistic pathways through which FMC exerts its effects on gut health and metabolic regulation.

## Data Availability

The data presented in the study are deposited in NCBI SRA repository, accession number PRJNA1196127.

## Footnotes

^1^ https://huttenhower.sph.harvard.edu/picrust/
