# Supplementary material for: Momoridica charantia and fermented Momoridica charantia with Leuconostoc mesenteroides MKSR change intestinal microbial diversity indices and compositions in high-fat and high-cholesterol diet-fed C57BL/6 male mice
Source: Front Vet Sci. 2024 Dec 17;11:1496067. doi: 10.3389/fvets.2024.1496067 (PMC11686596; doi:10.3389/fvets.2024.1496067)
Supplement: Supplementary file 1 [file Data_Sheet_1.docx]

**Supplementary files**

**
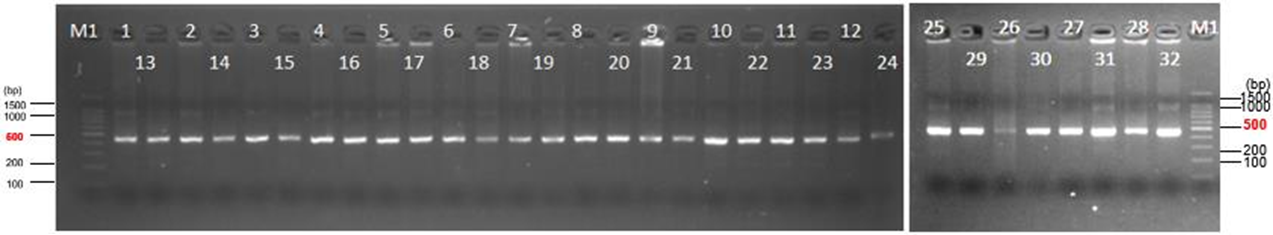
**

**Figure S1. PCR products gel electrophoresis images.** Three microliters of each sample (n=32) were loaded while 1 µL of sample ladder was loaded (and shown as M1 in the image).


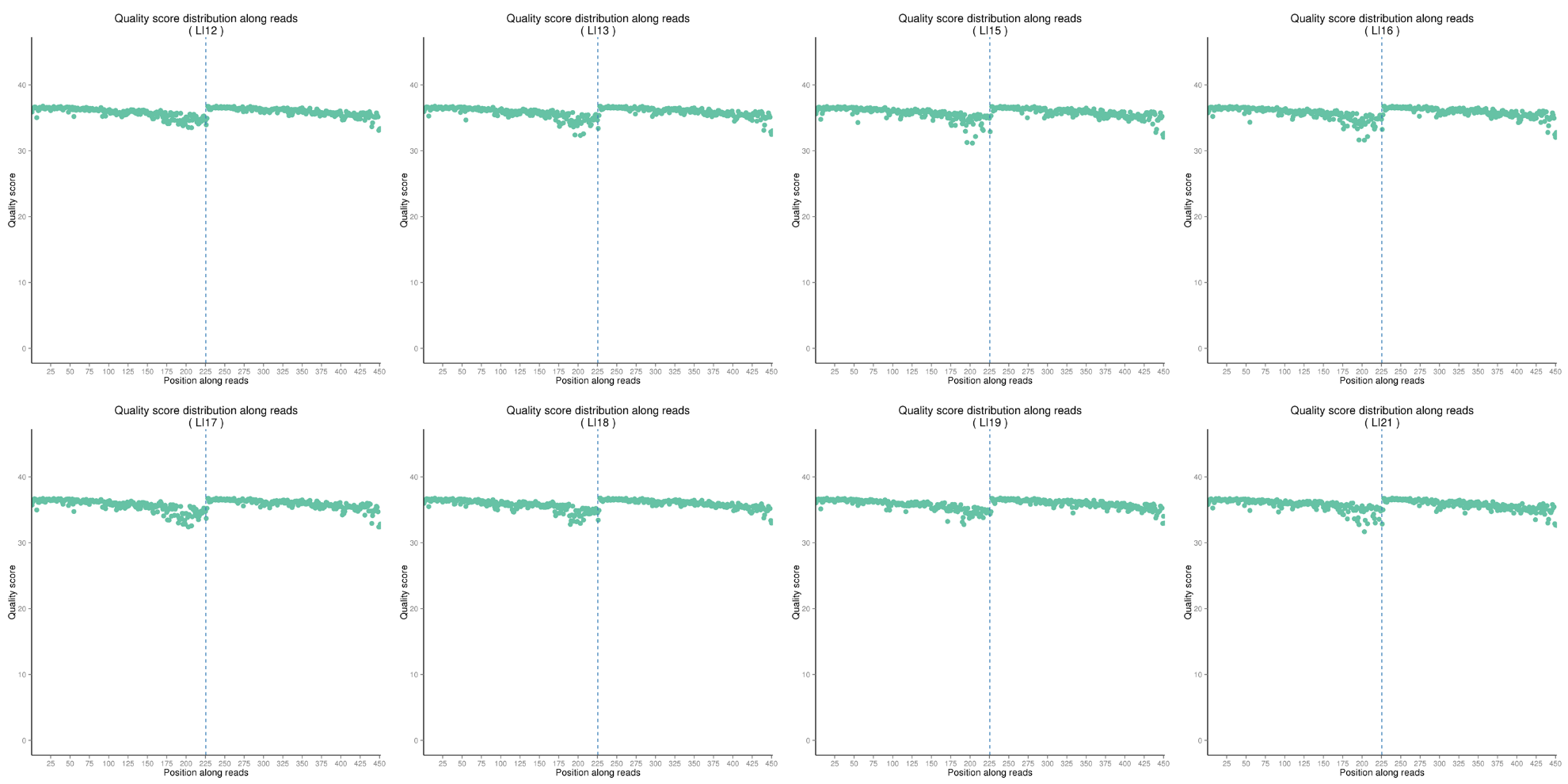


**Figure S2A. The sequencing error rate for the negative control group samples.**


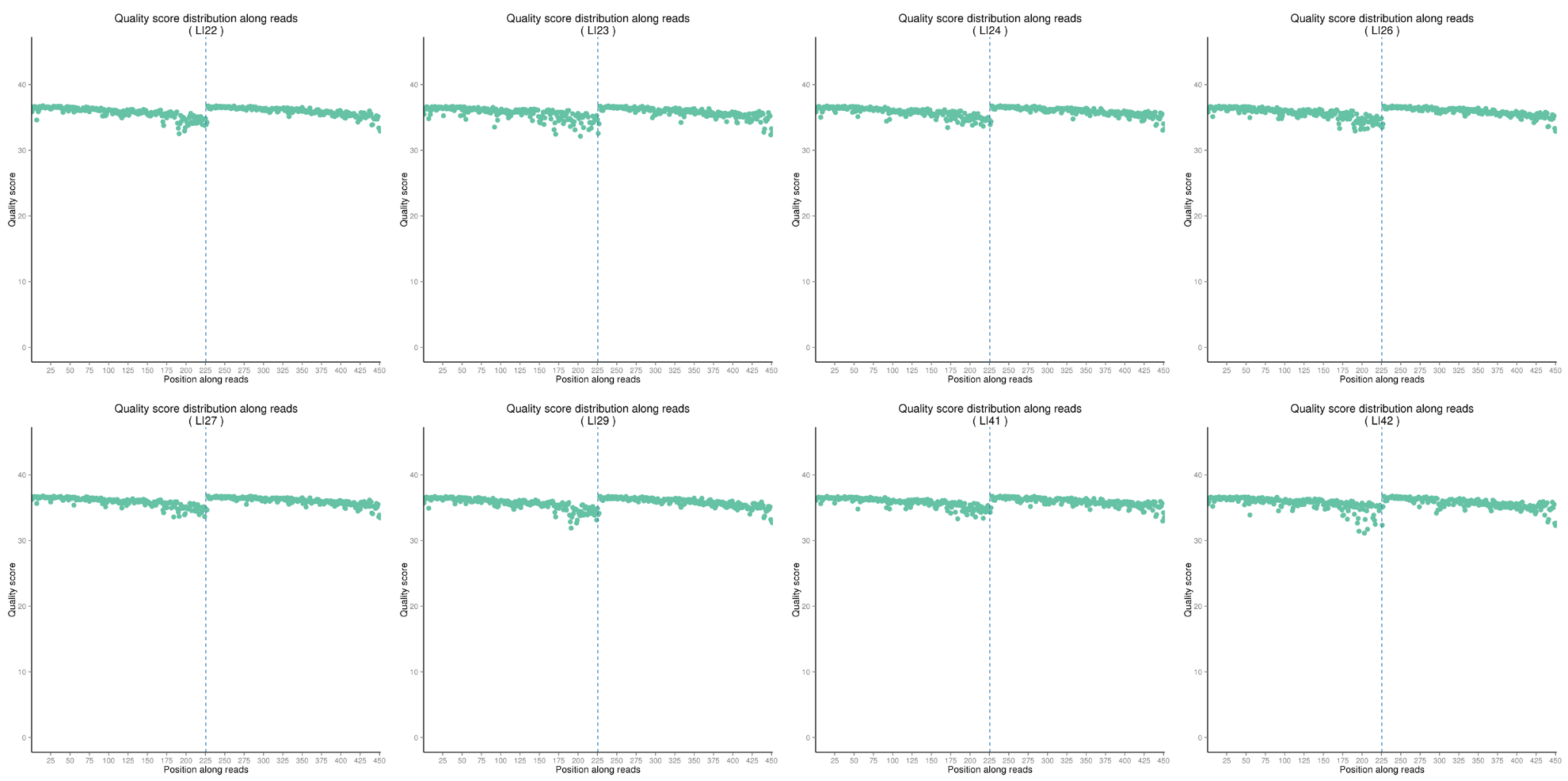


**Figure S2B. The sequencing error rate for the positive control group samples.**


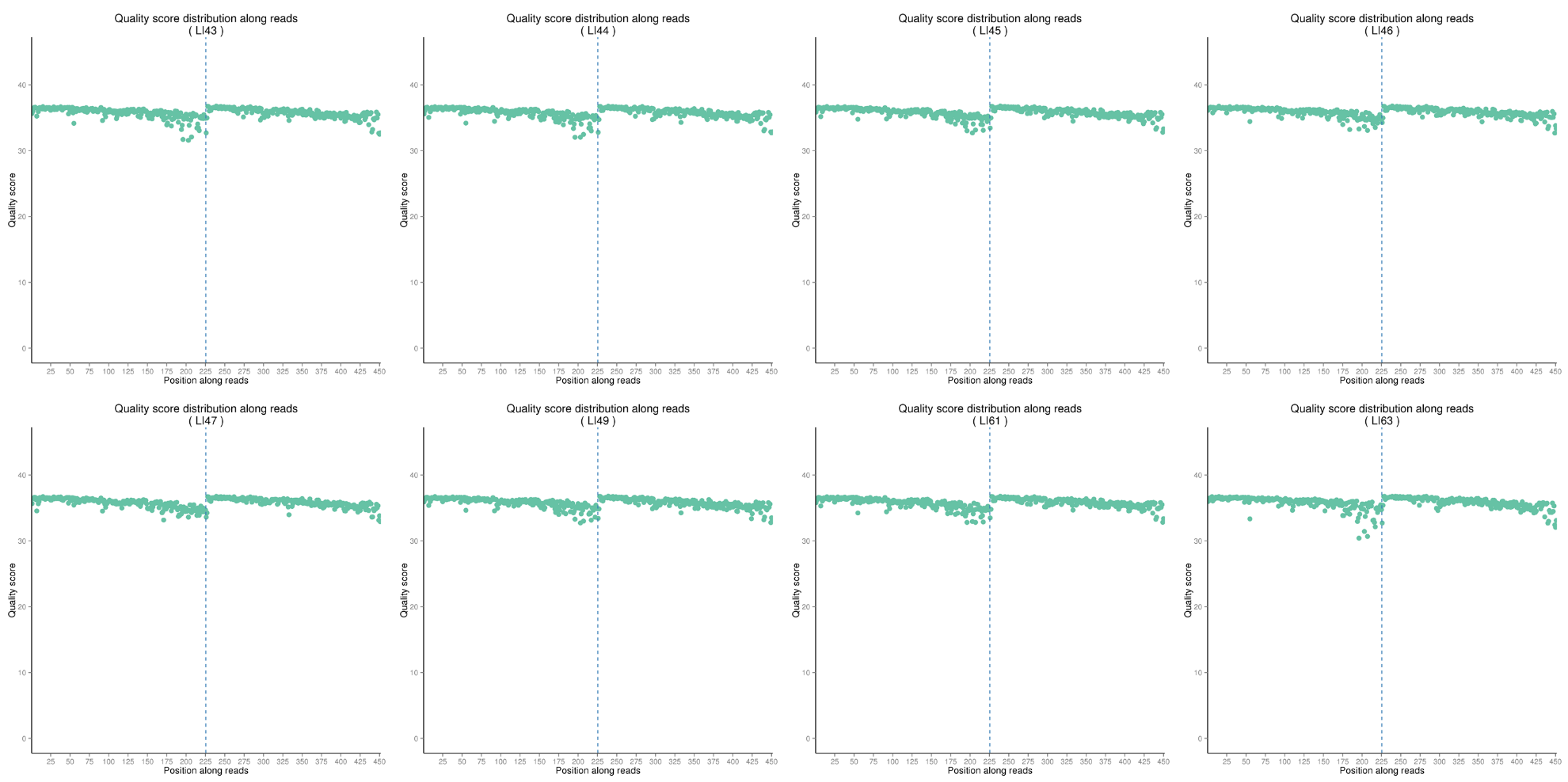


**Figure S2C. The sequencing error rate for the 4% MC group samples.**


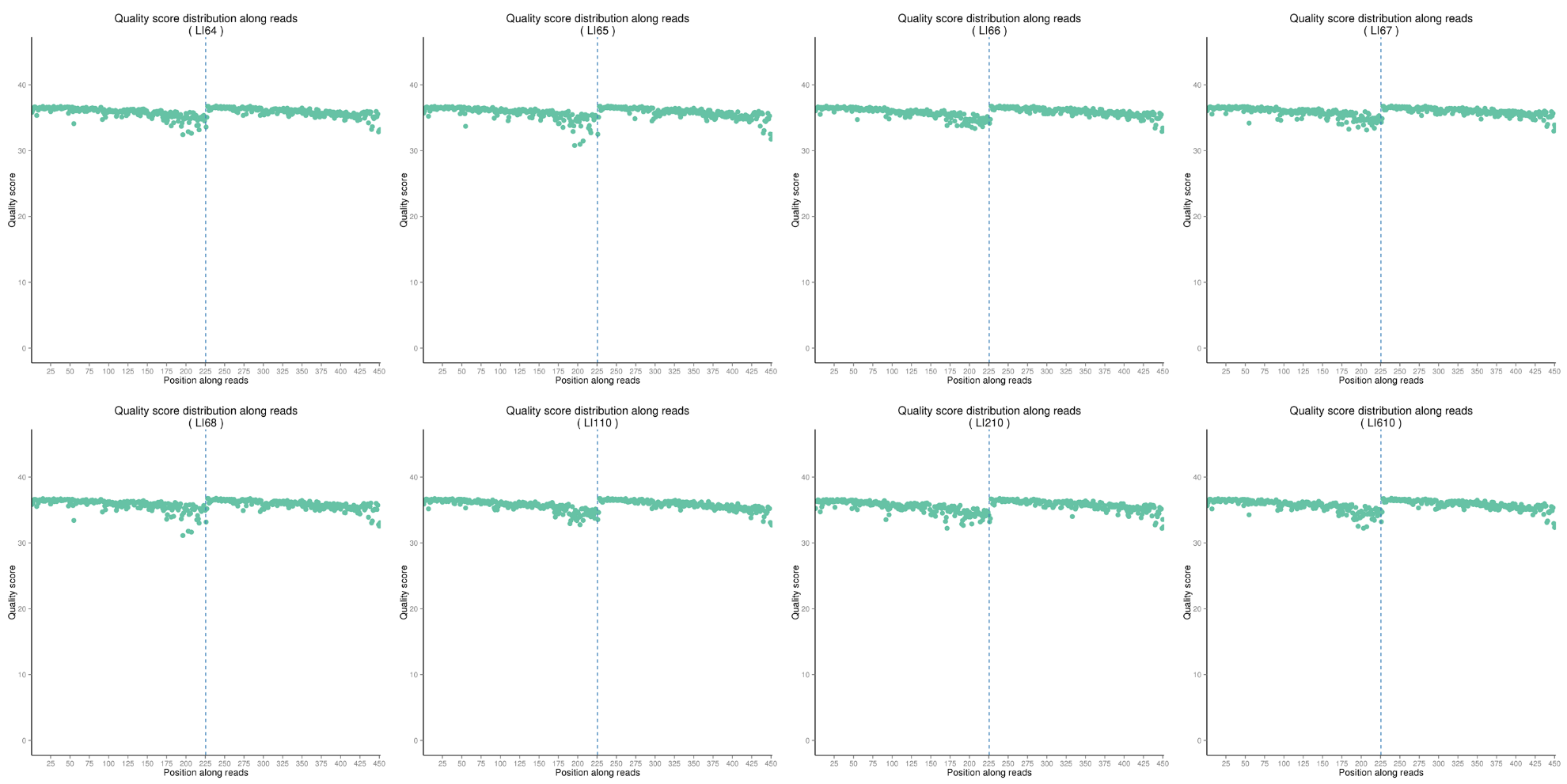


**Figure S2D. The sequencing error rate for the 4% FMC group samples.**

**Table S1. Phred score and error base**

| **Phred score** | **Error base** | **Right base** |
| --- | --- | --- |
| **10** | 1/10 | 90% |
| **20** | 1/100 | 99% |
| **30** | 1/1000 | 99.9% |
| **40** | 1/10000 | 99.99% |
